# Cognitive Ability Does Not Predict Objectively Measured Sedentary Behavior: Evidence From Three Older Cohorts

**DOI:** 10.1037/pag0000221

**Published:** 2018-03

**Authors:** Iva Čukić, Richard Shaw, Geoff Der, Sebastien F. M. Chastin, Manon L. Dontje, Jason M. R. Gill, John M. Starr, Dawn A. Skelton, Ratko Radaković, Simon R. Cox, Philippa M. Dall, Catharine R. Gale, Ian J. Deary

**Affiliations:** 1 Centre for Cognitive Ageing and Cognitive Epidemiology, Department of Psychology, University of Edinburgh; 2 MRC/CSO Social and Public Health Sciences Unit, University of Glasgow; 3 Centre for Cognitive Ageing and Cognitive Epidemiology, Department of Psychology, University of Edinburgh, and MRC/CSO Social and Public Health Sciences Unit, University of Glasgow; 4 Institute for Applied Health Research, School of Health and Life Sciences, Glasgow Caledonian University, and Department of Movement and Sports Sciences, Faculty of Medicine and Health Science, Ghent University; 5 Institute for Applied Health Research, School of Health and Life Sciences, Glasgow Caledonian University, and School of Population and Global Health, University of Western Australia; 6 Institute of Cardiovascular and Medical Sciences, University of Glasgow; 7 Centre for Cognitive Ageing and Cognitive Epidemiology, Department of Psychology, University of Edinburgh, and Alzheimer Scotland Dementia Research Centre, University of Edinburgh; 8 Institute for Applied Health Research, School of Health and Life Sciences, Glasgow Caledonian University; 9 Centre for Cognitive Ageing and Cognitive Epidemiology, Department of Psychology, University of Edinburgh, and Alzheimer Scotland Dementia Research Centre, University of Edinburgh; 10 Centre for Cognitive Ageing and Cognitive Epidemiology, Department of Psychology, University of Edinburgh; 11 Institute for Applied Health Research, School of Health and Life Sciences, Glasgow Caledonian University; 12 Centre for Cognitive Ageing and Cognitive Epidemiology, Department of Psychology, University of Edinburgh, and MRC Lifecourse Epidemiology Unit, University of Southampton; 13 Centre for Cognitive Ageing and Cognitive Epidemiology, Department of Psychology, University of Edinburgh

**Keywords:** sedentary behavior, cognitive ability, intelligence, activPAL, objective measures

## Abstract

Higher cognitive ability is associated with being more physically active. Much less is known about the associations between cognitive ability and sedentary behavior. Ours is the first study to examine whether historic and contemporaneous cognitive ability predicts objectively measured sedentary behavior in older age. Participants were drawn from 3 cohorts (Lothian Birth Cohort, 1936 [LBC1936] [*n* = 271]; and 2 West of Scotland Twenty-07 cohorts: 1950s [*n* = 310] and 1930s [*n* = 119]). Regression models were used to assess the associations between a range of cognitive tests measured at different points in the life course, with sedentary behavior in older age recorded over 7 days. Prior simple reaction time (RT) was significantly related to later sedentary time in the youngest, Twenty-07 1950s cohort (*p* = .04). The relationship was nonsignificant after controlling for long-standing illness or employment status, or after correcting for multiple comparisons in the initial model. None of the cognitive measures were related to sedentary behavior in either of the 2 older cohorts (LBC1936, Twenty-07 1930s). There was no association between any of the cognitive tests and the number of sit-to-stand transitions in any of the 3 cohorts. The meta-analytic estimates for the measures of simple and choice RT that were identical in all cohorts (*n* = 700) were also not significant. In conclusion, we found no evidence that objectively measured sedentary time in older adults is associated with measures of cognitive ability at different time points in life, including cognitive change from childhood to older age.

Sedentary behavior (SB) is defined as any waking activity characterized by low energy expenditure (≤1.5 metabolic equivalents) in a sitting or reclining posture (Barnes et al., 2012; Chastin, Schwarz, & Skelton, 2013). It has been identified as a risk factor for adverse health outcomes, such as diabetes and cardiovascular disease (Ford & Caspersen, 2012; Wilmot et al., 2012), as well as all-cause, cancer-, and cardiovascular-disease-specific mortality (Biswas et al., 2015; Katzmarzyk, Church, Craig, & Bouchard, 2009). Being less sedentary has been shown to be a predictor of successful aging in middle-aged and older adults independent of physical activity (de Rezende, Rey-López, Matsudo, & do Carmo Luiz, 2014; Dogra & Stathokostas, 2012). Sedentary behavior increases with age, and older adults are the most sedentary subpopulation (Davis et al., 2011; Harvey, Chastin, & Skelton, 2013, 2015; Matthews et al., 2008). It is estimated that time spent in sedentary activities in the adult population will increase to approximately 42 hr per week in the United States, and 52 hr per week in the United Kingdom, by 2030 (Ng & Popkin, 2012). The worldwide ageing of populations means that sedentary behavior is set to be increasingly prevalent, and thus the associated health burden will continue to increase (World Health Organization, 2011). It is therefore of particular importance to understand predictors of sedentary behavior in older age.

One well-established predictor of a wide range of healthy behaviors in older age, including physical activity, is cognitive ability (Batty, Deary, Schoon, & Gale, 2007; Deary, Weiss, & Batty, 2010; Hillman et al., 2006). Much less is known about the links, both cross-sectional and longitudinal, between cognitive ability and sedentary behavior. One study looked at cross-sectional associations of cognitive performance, operationalized as verbal memory and executive function, with sedentary activities (computer use and TV viewing) in older adults (Kesse-Guyot et al., 2012). The study found that better cognitive performance was cross-sectionally linked to more computer use but less TV viewing time. However, these measures of sedentary behavior were self-reported, and thus may not be accurate measures of sedentary time (Dall et al., 2017; Reilly, et al., 2008). Older adults often underestimate their sedentary time, as they can find it cognitively challenging to recollect and estimate (Harvey et al., 2015; van Uffelen, Heesch, Hill, & Brown, 2011), and a study showed that adults tended to underestimate their sedentary time by 2 to 4 hr per day (Chastin, Culhane, & Dall, 2014). The measures were also incomplete, as they did not cover the full range of possible sedentary activities.

One study examined cross-sectional associations between objectively measured sedentary behavior and cognitive status at Age 79 (Lord et al., 2011). Cognitive status was assessed using the Cambridge Neuropsychological Automated Testing Battery, the National Adult Reading Test, and the Mini Mental State Exam. None of the cognitive measures were significantly associated with the objectively measured sedentary time; however, the sample size was small (*n* = 56), and thus underpowered to detect modest associations.

The aim of the present study was to explore whether prior and contemporaneous cognitive ability is associated with objectively measured sedentary behavior in three large Scottish cohorts of older adults.

## Method

### Participants

Participants for the Seniors Understanding Sedentary Patterns (USP) study were recruited from three cohorts of the two Scottish longitudinal studies: The Lothian Birth Cohort, 1936 (LBC1936), and two cohorts of the West of Scotland Twenty-07 study (Twenty-07), that is, the 1930s and 1950s cohorts. Numbers in the cohorts’ titles represent the year or the decades of birth for LBC1936 and Twenty-07 cohorts, respectively. The same protocol for collecting sedentary behavior data was employed in all cohorts. The cognitive ability and other data from previous and current waves, when available, were used to investigate predictors of sedentary behavior in older age.

#### Lothian Birth Cohort, 1936

The LBC1936 participants were drawn from Wave 4 of the Lothian Birth Cohort, 1936—a follow-up study of the Scottish Mental Survey, 1947 (SMS1947). The fourth wave of assessment was conducted when participants were 79 years of age. All participants in the LBC1936 were community dwelling. Full details on the recruitment and testing procedures are provided elsewhere (Deary, Gow, Pattie, & Starr, 2012; Deary et al., 2007).

The target number of participants from the LBC1936 cohort for the Seniors USP study was 300. In total, the first 373 participants who attended Wave 4 were invited to take part until 304 agreed and had the physical activity monitor fitted, of whom 302 returned the monitor. There were no additional selection criteria to take part in the study. All participants provided written informed consent. Ethical approval was obtained from the Multi-Centre Research Ethics Committee for Scotland.

#### Twenty-07 Study (1930s and 1950s cohorts)

**Figure 1 fig1:**
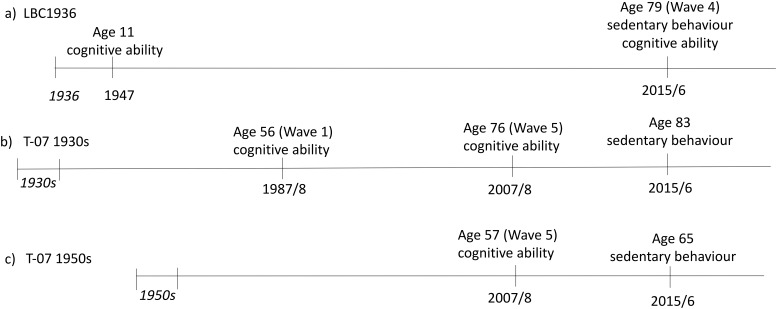
The timeline of data collection for the three cohorts included in the study. Numbers in cursive represent year or decades of birth. LBC1936 = Lothian Birth Cohort 1936; T-07 1930s = Twenty-07 1930s cohort; T-07 1950s = Twenty-07 1950s cohort.

The Twenty-07 study includes three cohorts born approximately 20 years apart, two of whom are included here: the 1930s and 1950s cohorts. Data for the main Twenty-07 study were collected in five waves between 1987 and 2008, and the sedentary behavior data were collected concurrently with Wave 4 of the LBC1936 for Seniors USP. All participants who lived in the greater Glasgow area were eligible to take part in the study. All eligible participants in the 1930s cohort (*n* = 468) were approached, of which 129 agreed to wear the activity monitor. A random sample of eligible people (*n* = 765) in the 1950s cohort was approached, of which 340 agreed to take part. Ethics approval for the Twenty-07 study was obtained from the NHS and/or Glasgow University Ethics Committees. Detailed descriptions of the Twenty-07 study design and data collection are provided elsewhere (Benzeval et al., 2009). Figure 1 shows comparative timelines of the three cohorts.[Fig-anchor fig1]

### Measures

#### Sedentary behavior

Sedentary behavior was measured as a 7-days continuous recording (7 × 24 hr, starting at midnight) using the activPAL activity monitor (activPAL3c; PAL Technologies, Glasgow, United Kingdom). The validity of the activPAL monitor has previously been established by comparing direct observations with the data recorded using the activPAL devices. Participants wore the device while being observed over two 6-hr periods. The recording underestimated the sitting time by <3% on average (Kozey-Keadle, Libertine, Lyden, Staudenmayer, & Freedson, 2011). The error rate for measuring activity at the treadmill or outdoors was <1% (Grant, Dall, Mitchell, & Granat, 2008). Furthermore, the accuracy of the activPAL measures was shown to be within ±5% of those obtained through commonly used video observations for physical activity and posture (Sellers, Dall, Grant, & Stansfield, 2016). Finally, the interdevice reliability between four devices was shown to be high (Sellers et al., 2016).

The activPAL device is small and light to wear (53 × 35 × 7 mm; 15 g). Participants had the monitor fitted to the front of the thigh of their dominant leg using a waterproofing dressing. They wore the monitor continuously for 7 full days including during sleep, bathing, or swimming. During the time they wore the monitor, participants also recorded the time they woke up and fell asleep each morning and evening. The activPAL is a triaxial inclinometer that continuously monitors the position of the thigh. Recorded data were downloaded using activPAL software (Version 7.2.32; PAL Technologies) and collated with sleep diary data in the R environment (R Core Team, 2016). The outcome measures derived in this fashion were the average percentage of waking time spent sedentary per day (hereafter, “sedentary time”) and the number of sit to stand transitions per each day of assessment (hereafter, “sit-to-stand transitions”). The protocol for obtaining the outcome measures was consistent across all three cohorts used in this study.

#### Cognitive ability

The Moray House Test No. 12 (MHT) was administered during the SMS1947 (Deary, Whalley, & Starr, 2009). The test consisted of a variety of items designed to assess reasoning ability—for example, word classification, analogies, reasoning, arithmetic, spatial items, and so forth. The MHT is a reliable and externally validated measure of general intelligence (Deary et al., 2007; Deary, Whiteman, Starr, Whalley, & Fox, 2004). The MHT scores are available at mean ages of 11 and 79 years in the LBC1936, corrected for age (in days) at the time of cognitive testing. Lifetime cognitive change was assessed as residuals of the regression models predicting Age 79 MHT score from Age 11 MHT scores (see Figure 1).

A General Cognitive Ability (*g*) factor was computed as the first unrotated principal component of the six tests taken from the Wechsler Adult Intelligence Scale—Third UK Edition (Wechsler, 1998). The tests used were Matrix Reasoning, Block Design, Letter-Number Sequencing, Symbol Search, Digit Span Backwards, and Digit Symbol. The *g* factor was calculated at Age 79 years in the LBC1936, the same wave of measurement sedentary behavior study was conducted.

Part 1 of the Alice Heim 4 test (AH4; Heim, 1970) was used to assess general intelligence in the Twenty-07 cohorts. The test comprises 32 items of verbal and 33 items of numerical ability, including series completion, mental arithmetic, vocabulary, and analogies. The test correlates highly with other measures of general ability such as Raven’s Progressive Matrices (Batty, Deary, Benzeval, & Der, 2010; Heim, 1970). The AH4 scores used here were from the most recent wave of measurement (Wave 5) of the 1950s cohort, and Waves 1 and 5 of the 1930s cohort. Cognitive change between Waves 1 and 5 (approximately 20 years apart) was assessed as residuals of the regression model predicting Wave 5 AH4 scores from Wave 1 AH4 scores in the 1930s cohort (see Figure 1 for the exact timelines of data collection).

Simple reaction time (SRT) mean and four-choice reaction time (CRT) mean were obtained with a commonly used box designed specifically for this purpose (Deary & Der, 2005; Deary, Der, & Ford, 2001). Both tasks had eight practice trials, as well as 20 and 40 tests trials for the SRT and CRT tasks, respectively. Participants who made 10 or more errors on the CRT (*n* = 3) or who had missing values on either of the tasks (*n* = 4) were removed from the analyses that included these variables. We used SRT and CRT scores from the most recent waves of measurement, which are Wave 4 of the LBC1936 (contemporaneous with sedentary behavior measures), and Wave 5 of the 1930s and 1950s Twenty-07 cohorts (about 8 years prior to sedentary behavior measures; Figure 1).

#### Covariates

The following variables were used as covariates: age at time of cognitive testing (continuous), sex (1 = men, 2 = women), maximum educational attainment (0 = no qualification, 1 = basic education including O levels and A levels, 2 = advanced education including semiprofessional and professional occupations, or a degree), employment (0 = employed, self-employed, or semiretired, 1 = retired), and long-standing illness. Illness was coded as 1 if the participant answered “yes” to both of the following questions: “Do you have any long-standing illness, disability or infirmity?” and “Does this condition limit your activities in any way?”; illness was coded as 0 if they answered “no” to either of the two questions. All covariates were consistent across all three cohorts.

### Statistical Analysis

Comparisons between the sexes were done using *t* tests for continuous variables and chi-square tests for categorical variables. Linear regression models were used to assess the associations between cognitive ability and measures of sedentary behavior. The dependent variables were average percent of waking time spent sedentary per day and the average number of sit-to-stand transitions per day. The number of sit-to-stand transitions measure was positively skewed in all three cohorts and was therefore square-root transformed. We ran a series of regression models for each of the two sedentary behavior outcomes. In Model 1, a measure of cognitive ability was entered as an independent variable, controlling for age and sex. In Model 2, education was added as another independent variable. In Model 3, a measure of long-standing illness was added to the previous model as independent variable. These steps were repeated for each of the cognitive measures used in the study, including measures of cognitive change. In the youngest cohort only, Model 4 was performed to additionally control for employment status. This was not possible with the two older cohorts, as the number of employed participants was too low (11 in LBC1936, four in Twenty-07 1930s cohort). Given the large number of statistically dependent tests performed, we controlled for multiple testing using the false discovery rate test (FDR) within each of the analyzed cohorts (Benjamini & Yekutieli, 2001). All analyses were conducted using R (Version 3.3.1; R Core Team, 2016).

## Results

### Descriptive Statistics

Descriptive statistics for all variables in the study are presented in Table 1. The oldest cohort, the Twenty-07 1930s cohort, with a mean age of 83 years, was the most sedentary cohort, with approximately 68% of the waking time spent in sedentary behavior. LBC1936 was second, with a mean age of 79 years and approximately 65% of the waking time spent sedentary. Finally, the youngest, the Twenty-07 1950s cohort, had a mean age of 65 years, and spent approximately 62% of the waking time engaged in sedentary behavior (see Table 1 for more details). Men and women showed similar levels of sedentary behavior in both Twenty-07 cohorts, but males were more sedentary than females in the LBC1936. Mean levels of cognitive ability were similar for men and women in all three cohorts (see Table 1). At the zero-order level, two correlations were significant: the association between sex and total sedentary time in the LBC1936 cohort (*r* = −.23, *p* < .001), and the association between long-standing illness and total sedentary time in the 1950s Twenty-07 cohort (*r* = .29, *p* < .001). None of the other variables in the study were significantly related to either of the two sedentary behavior variables at the zero-order level (*p*s ranging between .26 and .99).[Table-anchor tbl1]

### Cognitive Ability and Sedentary Time

First, we examined the associations between cognitive measures and sedentary time in the youngest, Twenty-07 1950s cohort (see Table 2). SRT measured in Wave 5 was significantly and positively associated with sedentary time in Model 1 (standardized β = .12) but not in any of the models including additional controls. The reduction in effect size was small. There were no significant associations between AH4 measures or CRT measures from Wave 5 with sedentary time in the 1950s cohort. In the analyses of data from the Twenty-07 1930s cohort and the LBC1936, none of the cognitive measures (including measures of cognitive change between childhood and adulthood, and change within older adulthood) were significantly associated with sedentary time (see Table 2 for the full list of estimates).[Table-anchor tbl2]

### Cognitive Ability and Sit-to-Stand Transitions

The next set of models examined whether cognitive measures related to the number of sit-to-stand transitions. The results were consistently null: None of the cognitive measures in any of the three cohorts were significantly associated with the number of sit-to-stand transitions (see Table 3).[Table-anchor tbl3]

### Correction for Multiple Testing

Lastly, because of a large number of dependent tests we ran, to address the problem of multiple testing, we corrected observed *p* values using the FDR test (Benjamini & Yekutieli, 2001). This was done for the number of tests within each cohort separately. After doing so, none of the *p* values reached standard significance thresholds. Therefore, the initially observed significant association between SRT and sedentary behavior in the 1950s cohort could be considered a Type I error.

### Meta-Analysis of the SRT and CRT Measures

**Figure 2 fig2:**
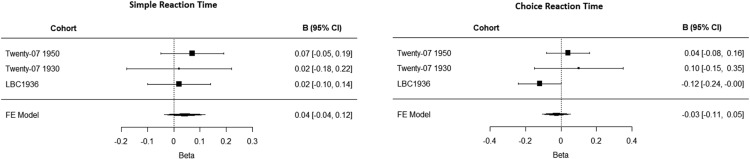
The meta-analytic estimates for the measures of simple reaction time and choice reaction time predicting total sedentary time, controlling for age, sex, education, and long-standing illness (Model 3 in Table 2).

Even though we utilize data from three longitudinal studies, the number of participants with all available data ranges from 119 to 309 across cohorts, with 80% power to detect a correlation of the magnitude ranging between 0.25 and 0.16 (low to moderate, according to Cohen’s conventions). If the real effect size of the cognitive-ability/sedentary-behavior association is smaller, our samples would not have the sufficient power to detect it (Cohen, 1992). To address this, we meta-analyzed the results for the measures of SRT and CRT, as they were assessed using an identical method in all three cohorts (*n* = 700). The meta-analytic estimate for SRT was β = .04, *p* = .30, and for CRT was β = −.003, *p* = .53, for the fully adjusted models predicting total sedentary time (see Figure 2). The meta-analytic estimates were similar for fully adjusted models predicting sit-to-stand transitions (β = −.04, *p* = .33 for SRT; β = .04, *p* = .08 for CRT). In addition, we conducted a combined analysis with participants nested within cohorts, and fitted multilevel models corresponding to those reported in the meta-analysis previously. In the model predicting total sedentary time that included the random effect of cohort, and fixed effects of SRT, adjusted for all covariates, the effect of SRT was 0.07 (*t* = 0.743). Similarly, in the model predicting total sedentary time that included the random effect of cohort, and fixed effects of CRT and all covariates, the effect of CRT was 0.03 (*t* = 0.306). Jointly, these results suggest that it is unlikely that our failure to detect an effect could be explained by the lack of statistical power.[Fig-anchor fig2]

### Validity of Sedentary Behavior Measures

To rule out the possibility that our results are nonsignificant not because of the true lack of the association between cognitive ability and sedentary behavior, but because the measures of sedentary behavior we used are not reliable or valid measures of real sedentary behaviors, we tested whether they correlate in an expected way with other relevant measures available in the LBC1936 cohort. Total sedentary time correlated in an expected way with current BMI (*r* = .30, *p* < .001). The self-reported physical activity was available from a previous wave approximately 9 years prior to the activPAL assessment. Total sedentary time did not significantly correlate with this measure; however, the activPAL measure of step-count, and therefore physical activity, did (*r* = .32, *p* < .001). Finally, we observed the difference in total sedentary time between the youngest and the oldest cohort, namely, in the youngest, Twenty-07 1950s cohort with the average age of 65, the average sedentary time was 60% of the waking hours. On the other hand, in the oldest, Twenty-07 1930s cohort, with the average age of 83, the average sedentary time was 68%, as expected (see Table 1). Based on these associations and differences, we conclude that our lack of significant associations is not likely a result of insufficient reliability and validity of sedentary behavior measures.

## Discussion

The present study examined the associations between a broad range of cognitive tests, taken from multiple time points, and objectively measured sedentary behavior in older age in three Scottish cohorts. None of the cognitive measures were associated with current or future sedentary time after controlling for highest educational qualifications and present long-standing illness. Similarly, none of the cognitive measures in any of the cohorts were associated with the number of daily sit-to-stand transitions. Furthermore, SRT measure that was nominally significantly associated with sedentary behavior in Model 1 in the youngest cohort did not sustain correction for the false discovery rate. The meta-analytic estimates for the measures of SRT and CRT across all three cohorts were also nonsignificant. In summary, we found no evidence that prior or contemporaneous cognitive ability or cognitive change are associated with objectively measured sedentary behavior in older age.

Our results are not in line with a study that showed cognitive status is related to different aspects of sedentary time (Kesse-Guyot et al., 2012). One explanation could be that, although Kesse-Guyot and colleagues (2012) reported differing associations between different aspects of sedentary behavior and cognitive performance, in our study, we used a measure of total sedentary time that could not distinguish between these different activities. It is possible that cognitive ability has positive associations with certain sedentary behaviors but negative associations with others (Kesse-Guyot et al., 2012), which would cancel out and yield a negative association with a measure of total sedentary time. Another possibility is that the discrepancy in the findings results from different nature of the measures. Kesse-Guyot et al. used self-reported measures of sedentary behavior, which may be biased (Dall et al., 2017; Reilly et al., 2008), and our sedentary measures were objective. Social desirability, or perhaps other health behaviors associated with sedentary behavior, may influence self-report.

On the other hand, our results are consistent with that of a small study that used the same outcome measure, and found no association between several measures of cognitive performance and objectively measured total sedentary time in a sample of older adults (Lord et al., 2011).

Our study has several strengths. We were able to use a variety of cognitive tests that include general ability and processing speed measures. Furthermore, we had access to cognitive data from multiple time points, starting from childhood to several waves of follow-up in older age. This allowed us to test whether prior and contemporaneous cognitive ability, as well as cognitive change, relate to measures of sedentary behavior. Next, we used measures of sedentary behavior recorded objectively over a period of 7 days. This increases reliability and validity of the outcome measures over most of the previous studies that relied on self-reports and proxy measures (e.g., screen time). Finally, we were able to test these associations in three ageing cohorts, which shared identical measures of sedentary behavior as well as some of the cognitive measures. This allowed for direct replication and comparison of the models, thus providing us with more reliable and generalizable results.

The study also had some limitations. Primarily, measures of sedentary behavior were available only in the most recent wave of assessment, whereas addressing any direction of the observed effects would also require measures of sedentary behavior taken in previous waves. Importantly, we were not able to assess whether prior sedentary behavior was associated with subsequent cognitive functioning. Finally, we used a measure of total time spent in sedentary activities, but we were not able to distinguish between different sedentary activities that could have differential associations with cognitive ability (Kesse-Guyot et al., 2012).

In conclusion, we found no evidence that prior or current cognitive ability is associated with objectively measured sedentary time in older age. Future studies should aim to replicate these findings using larger samples and multiple waves of objectively measured sedentary behavior, and taking into account different types of sedentary behavior.

## Figures and Tables

**Table 1 tbl1:** Descriptive Statistics of the Three Cohorts Used in the Study Stratified by Sex

Variables	Males	Females	*p* value for difference	Total
Twenty-07 1950s	*n* = 145	*n* = 165		*n* = 310
	*M* (*SD*)	*M* (*SD*)		*M* (*SD*)
Age^a^	64.64 (1.02)	64.53 (.78)	.31	64.58 (.90)
Sedentary time (%)	61.60 (10.08)	60.17 (11.34)	.24	60.84 (10.77)
Sit-to-stand (number)	48.70 (12.80)	49.48 (14.34)	.61	49.12 (13.63)
AH4 Wave 5	38.04 (11.51)	36.43 (10.09)	.19	37.17 (10.78)
SRT Wave 5	286.72 (47.75)	296.41 (78.76)	.20	291.88 (66.17)
CRT Wave 5	616.43 (91.62)	618.25 (128.64)	.89	617.39 (112.50)
	*n* (%)	*n* (%)		*n* (%)
Education^a^			.02	
Low	8 (5.5)	17 (10.3)		25 (8.1)
Medium	87 (60.0)	74 (44.8)		161 (51.9)
High	50 (34.5)	74 (44.8)		124 (40.0)
Illness^a^	24 (16.6)	36 (21.8)	.30	60 (19.4)
Employed^a^	70 (48.3)	39 (23.6)	<.001	109 (35.2)
Twenty-07 1930s	*n* = 54	*n* = 65		*n* = 119
	*M* (*SD*)	*M* (*SD*)		*M* (*SD*)
Age^a^	83.32 (.68)	83.46 (.57)	.21	83.40 (.62)
Sedentary time (%)	67.95 (11.98)	68.46 (10.03)	.80	68.23 (10.92)
Sit-to-stand (number)	41.40 (11.28)	43.93 (15.20)	.31	42.78 (13.57)
AH4 Wave 1	32.71 (9.66)	32.89 (10.36)	.92	32.81 (10.01)
AH4 Wave 5	28.71 (9.17)	29.54 (10.71)	.66	29.17 (10.01)
SRT Wave 5	326.42 (102.37)	348.83 (108.72)	.28	337.60 (102.40)
CRT Wave 5	718.45 (103.65)	719.83 (91.53)	.95	719.25 (96.07)
	*n* (%)	*n* (%)		*n* (%)
Education^a^			.20	
Low	11 (20.4)	23 (35.4)		34 (28.6)
Medium	31 (57.4)	30 (46.2)		61 (51.3)
High	12 (22.2)	12 (18.5)		24 (20.2)
Illness^a^	19 (35.2)	32 (49.2)	.18	51 (42.9)
Employed^a^	1 (1.9)	3 (4.6)	.78	4 (3.4)
LBC1936	*n* = 140	*n* = 131		*n* = 271
	*M* (*SD*)	*M* (*SD*)		*M* (*SD*)
Age^a^	79.04 (.46)	78.90 (.41)	.01	78.97 (.44)
Sedentary time (%)	64.78 (9.79)	60.09 (10.48)	<.001	62.51 (10.38)
Sit-to-stand (number)	43.82 (12.46)	44.14 (10.40)	.08	43.97 (11.49)
Age 11 MHT	53.09 (17.34)	54.12 (14.28)	.06	53.59 (15.91)
Age 79 MHT^a^	63.42 (11.42)	64.32 (8.60)	.05	63.86 (10.12)
Age 79 CRT^a^	.72 (.12)	.68 (.10)	.01	.70 (.11)
Age 79 SRT^a^	.29 (.05)	.29 (.05)	.06	.29 (.05)
Age 79 *g*^a^	.05 (1.06)	−.00 (.91)	.66	.03 (.99)
	*n* (%)	*n* (%)		*n* (%)
Education^a^			.01	
Low	24 (17.1)	12 (9.2)		36 (13.3)
Medium	57 (40.7)	76 (58.0)		133 (49.1)
High	59 (42.1)	43 (32.8)		102 (37.6)
Illness^a^	24 (17.1)	25 (19.1)	.80	49 (18.1)
Employed^a^	4 (2.9)	7 (5.4)	.46	11 (4.1)
*Note*. AH4 = Alice Heim 4; MHT = Moray House Test; SRT = simple reaction time; CRT = choice reaction time; LBC1936 = Lothian Birth Cohort, 1936.				
^a^ Measured at the same time as sedentary behavior measurement.				

**Table 2 tbl2:** Standardized Betas (95% CIs) for the Models Assessing Relationships Between Lifetime Measures of Cognitive Ability and Objectively Measured Total Sedentary Time (Daily Average) in Older Age in Three Scottish Cohorts

Variables	Model 1		Model 2		Model 3		Model 4	
	β [95% CI]	*p*	β [95% CI]	*p*	β [95% CI]	*p*	β [95% CI]	*p*
Twenty-07 1950s								
AH4 Wave 5	−.08 [−.04, .20]	.18	−.08 [−022., .06]	.23	−.06 [−.20, .08]	.40	−.06 [−.20, .08]	.39
Simple RT Wave 5	.12 [.00, .24]	.04	.11 [−.01, .21]	.05	.07 [−.05, .19]	.21	.06 [−.06, .18]	.26
Choice RT Wave 5	.09 [−.03, .21]	.13	.08 [−.04, .20]	.17	.04 [−.08, .16]	.43	.05 [−.07, .17]	.41
Twenty-07 1930s								
AH4 Wave 1	−.08 [−.28, .12]	.41	−.08 [−.30, .14]	.47	−.08 [−.30, .14]	.45		
AH4 Wave 5	−.07 [−.27, .13]	.49	−.04 [−.26, .18]	.75	−.04 [−.26, .18]	.75		
AH4 change Wave 1–5	−.01 [−.21, .19]	.88	.02 [−.18, .22]	.91	.03 [−.17, .23]	.80		
Simple RT mean Wave 5	.04 [−.14, .22]	.70	.02 [−.18, .22]	.83	.02 [−.18, .22]	.82		
Choice RT mean Wave 5	.10 [−.14, .34]	.42	.12 [−.12, .36]	.33	.10 [−.15, .35]	.42		
LBC1936								
MHT Age 11	−.05 [−.17, .07]	.38						
MHT change Age 11–79	−.09 [−.21, .03]	13	−.06 [−.20, .08]	.33	−.06 [−.20, .08]	.34		
*g* Wave 4	−.06 [−.18, .06]	.36	−.02 [−.16, .12]	.81	−.01 [−.15, .13]	.90		
Simple RT Wave 4	.03 [−.09, .15]	.61	.02 [−.10, .14]	.73	.02 [−.10, .14]	.77		
Choice RT Wave 4	−.10 [−.22, .02]	.09	−.12 [−.24, 1.00]	.06	−.12 [−.24, 1.00]	.05		
*Note*. Model 1 = + age, sex; Model 2 = Model 1 + education; Model 3 = Model 2 + long-standing illness; Model 4 = Model 3 + employment status. The *p* values in the table are not corrected for multiple testing. None of the *p* values remain significant after false discovery rate correction (see main text for details). AH4 = Alice Heim 4; MHT = Moray House Test; SRT = simple reaction time; CRT = choice reaction time; LBC1936 = Lothian Birth Cohort, 1936.								

**Table 3 tbl3:** Standardized Betas (95% CIs) for the Models Assessing Relationships Between Lifetime Measures of Cognitive Ability and Objectively Measured Number of Sit to Stand Transitions (Daily Average) in Older Age in Three Scottish Cohorts

Variables	Model 1		Model 2		Model 3		Model 4	
	β [95% CI]	*p*	β [95% CI]	*p*	β [95% CI]	*p*	β [95% CI]	*p*
Twenty-07 1950s								
AH4 Wave 5	.05 [−.07, .17]	.37	.11 [−.03, .25]	.11	.11 [−.03, .25]	.12	.11 [−.03, .25]	.11
Simple RT Wave 5	−.06 [−.18, .06]	.27	−.07 [−.19, .05]	.20	−.07 [−.19, .05]	.22	−.06 [−.06, .18]	.29
Choice RT Wave 5	−.04 [−.16, .08]	.49	−.05 [−.17, .07]	.42	−.04 [−.16, .08]	.45	−.05 [−.17, .07]	.43
Twenty–07 1930s								
AH4 Wave 1	.08 [−.10, .26]	.41	.13 [−.09, .35]	.23	.13 [−.09, .35]	.24		
AH4 Wave 5	.05 [−.15, .25]	.60	.10 [−.12, .32]	.40	.10 [−.12, .32]	.41		
AH4 change Wave 1–5	.04 [−.14, .22]	.66	.05 [−.15, .20]	.63	.05 [−.15, .20]	.64		
Simple RT mean Wave 5	−.07 [−.27, .13]	.47	−.09 [−.29, .11]	.39	−.09 [−.29, .11]	.39		
Choice RT mean Wave 5	.09 [−.09, .27]	.32	.04 [−.21, −.29]	.78	.04 [−.21, −.29]	.77		
LBC1936								
MHT Age 11	.07 [−.05, .19]	.24						
MHT change Age 11–79	.03 [−.11, .14]	.99	−.03 [−.17, .11]	.65	.01 [−.01, .03]	.99		
*g* Wave 4	.02 [−.10, .14]	.80	−.04 [−.18, .10]	.60	−.04 [−.18, .10]	.61		
Simple RT Wave 4	.00 [−.12, .12]	.99	.01 [−.11, .13]	.86	.01 [−.11, .13]	.89		
Choice RT Wave 4	−.03 [−.15, .09]	.60	−.03 [−.15, .09]	.66	.03 [−.15, .09]	.60		
*Note*. Model 1 = + age, sex; Model 2 = Model 1 + education; Model 3 = Model 2 + long-standing illness; Model 4 = Model 3 + employment status. The *p* values in the table are not corrected for multiple testing. AH4 = Alice Heim 4; MHT = Moray House Test; SRT = simple reaction time; CRT = choice reaction time; LBC1936 = Lothian Birth Cohort, 1936.								

## References

[c1] Barnes J., Behrens T. K., Benden M. E., Biddle S., Bond D., Brassard P., . . . Colley R. (2012). Letter to the editor: Standardized use of the terms “sedentary” and “sedentary behaviours.” Applied Physiology Nutrition and Metabolism, 37, 540–542. 10.1139/h2012-024 22540258

[c2] Batty G. D., Deary I. J., Benzeval M., & Der G. (2010). Does IQ predict cardiovascular disease mortality as strongly as established risk factors? Comparison of effect estimates using the West of Scotland Twenty-07 cohort study. European Journal of Cardiovascular Prevention and Rehabilitation, 17, 24–27. 10.1097/HJR.0b013e328321311b 20101181

[c3] Batty G. D., Deary I. J., Schoon I., & Gale C. R. (2007). Childhood mental ability in relation to food intake and physical activity in adulthood: The 1970 British Cohort Study. Pediatrics, 119, e38–e45. 10.1542/peds.2006-1831 17200256

[c4] Benjamini Y., & Yekutieli D. (2001). The control of the false discovery rate in multiple testing under dependency. Annals of Statistics, 29, 1165–1188.

[c5] Benzeval M., Der G., Ellaway A., Hunt K., Sweeting H., West P., & Macintyre S. (2009). Cohort profile: West of Scotland twenty-07 study: Health in the community. International Journal of Epidemiology, 38, 1215–1223. 10.1093/ije/dyn213 18930962 PMC2935558

[c6] Biswas A., Oh P. I., Faulkner G. E., Bajaj R. R., Silver M. A., Mitchell M. S., & Alter D. A. (2015). Sedentary time and its association with risk for disease incidence, mortality, and hospitalization in adults: A systematic review and meta-analysis. Annals of Internal Medicine, 162, 123–132. 10.7326/M14-1651 25599350

[c7] Chastin S. F. M., Culhane B., & Dall P. M. (2014). Comparison of self-reported measure of sitting time (IPAQ) with objective measurement (activPAL). Physiological Measurement, 35, 2319–2328. 10.1088/0967-3334/35/11/2319 25341050

[c8] Chastin S. F. M., Schwarz U., & Skelton D. A. (2013). Development of a consensus taxonomy of sedentary behaviors (SIT): Report of Delphi Round 1. PLoS ONE, 8(12), e82313 10.1371/journal.pone.0082313 24312653 PMC3847079

[c9] Cohen J. (1992). A power primer. Psychological Bulletin, 112, 155–159. 19565683 10.1037//0033-2909.112.1.155

[c10] Dall P. M., Coulter E. H., Fitzsimons C. F., Skelton D. A., Chastin S., & Seniors USP Team (2017). Taxonomy of Self-Reported Sedentary Behaviour Tools (TASST) framework for development, comparison and evaluation of self-report tools: Content analysis and systematic review. British Medical Journal Open, 7, e013844 10.1136/bmjopen-2016-013844 PMC5775464 28391233

[c11] Davis M. G., Fox K. R., Hillsdon M., Sharp D. J., Coulson J. C., & Thompson J. L. (2011). Objectively measured physical activity in a diverse sample of older urban UK adults. Medicine and Science in Sports and Exercise, 43, 647–654. 10.1249/MSS.0b013e3181f36196 20689449

[c12] Deary I. J., & Der G. (2005). Reaction time, age, and cognitive ability: Longitudinal findings from age 16 to 63 years in representative population samples. Aging, Neuropsychology, and Cognition, 12, 187–215. 10.1080/13825580590969235

[c13] Deary I. J., Der G., & Ford G. (2001). Reaction times and intelligence differences: A population-based cohort study. Intelligence, 29, 389–399. 10.1016/S0160-2896(01)00062-9

[c14] Deary I. J., Gow A. J., Pattie A., & Starr J. M. (2012). Cohort profile: The Lothian Birth Cohorts of 1921 and 1936. International Journal of Epidemiology, 41, 1576–1584. 10.1093/ije/dyr197 22253310

[c15] Deary I. J., Gow A. J., Taylor M. D., Corley J., Brett C., Wilson V., . . . Starr J. M. (2007). The Lothian Birth Cohort 1936: A study to examine influences on cognitive ageing from age 11 to age 70 and beyond. BMC Geriatrics, 7, 28 10.1186/1471-2318-7-28 18053258 PMC2222601

[c17] Deary I. J., Weiss A., & Batty G. D. (2010). Intelligence and personality as predictors of illness and death: How researchers in differential psychology and chronic disease epidemiology are collaborating to understand and address health inequalities. Psychological Science in the Public Interest, 11, 53–79. 10.1177/1529100610387081 26168413

[c18] Deary I. J., Whalley L. J., & Starr J. M. (2009). The Scottish Mental Surveys of 1932 and 1947. Washington, DC: American Psychological Association 10.1037/11857-001

[c19] Deary I. J., Whiteman M. C., Starr J. M., Whalley L. J., & Fox H. C. (2004). The impact of childhood intelligence on later life: Following up the Scottish mental surveys of 1932 and 1947. Journal of Personality and Social Psychology, 86, 130–147. 10.1037/0022-3514.86.1.130 14717632

[c20] de Rezende L. F. M., Rey-López J. P., Matsudo V. K. R., & do Carmo Luiz O. (2014). Sedentary behavior and health outcomes among older adults: A systematic review. BMC Public Health, 14, 333 10.1186/1471-2458-14-333 24712381 PMC4021060

[c21] Dogra S., & Stathokostas L. (2012). Sedentary behavior and physical activity are independent predictors of successful aging in middle-aged and older adults. Journal of Aging Research, 2012, 190654 10.1155/2012/190654 22997579 PMC3446656

[c22] Ford E. S., & Caspersen C. J. (2012). Sedentary behaviour and cardiovascular disease: A review of prospective studies. International Journal of Epidemiology, 41, 1338–1353. 10.1093/ije/dys078 22634869 PMC4582407

[c23] Grant P. M., Dall P. M., Mitchell S. L., & Granat M. H. (2008). Activity-monitor accuracy in measuring step number and cadence in community-dwelling older adults. Journal of Aging and Physical Activity, 16, 201–214. 10.1123/japa.16.2.201 18483442

[c24] Harvey J. A., Chastin S. F., & Skelton D. A. (2013). Prevalence of sedentary behavior in older adults: A systematic review. International Journal of Environmental Research and Public Health, 10, 6645–6661. 10.3390/ijerph10126645 24317382 PMC3881132

[c25] Harvey J. A., Chastin S. F. M., & Skelton D. A. (2015). How sedentary are older people? A systematic review of the amount of sedentary behavior. Journal of Aging and Physical Activity, 23, 471–487. 10.1123/japa.2014-0164 25387160

[c27] Heim A. (1970). Manual for the AH4 Group Test of General Intelligence. Windsor, UK: NFER.

[c28] Hillman C. H., Motl R. W., Pontifex M. B., Posthuma D., Stubbe J. H., Boomsma D. I., & de Geus E. J. (2006). Physical activity and cognitive function in a cross-section of younger and older community-dwelling individuals. Health Psychology, 25, 678–687. 10.1037/0278-6133.25.6.678 17100496

[c29] Katzmarzyk P. T., Church T. S., Craig C. L., & Bouchard C. (2009). Sitting time and mortality from all causes, cardiovascular disease, and cancer. Medicine and Science in Sports and Exercise, 41, 998–1005. 10.1249/MSS.0b013e3181930355 19346988

[c30] Kesse-Guyot E., Charreire H., Andreeva V. A., Touvier M., Hercberg S., Galan P., & Oppert J. M. (2012). Cross-sectional and longitudinal associations of different sedentary behaviors with cognitive performance in older adults. PLoS ONE, 7(10), e47831 10.1371/journal.pone.0047831 23082222 PMC3474738

[c31] Kozey-Keadle S., Libertine A., Lyden K., Staudenmayer J., & Freedson P. S. (2011). Validation of wearable monitors for assessing sedentary behavior. Medicine and Science in Sports and Exercise, 43, 1561–1567. 10.1249/MSS.0b013e31820ce174 21233777

[c32] Lord S., Chastin S. F. M., McInnes L., Little L., Briggs P., & Rochester L. (2011). Exploring patterns of daily physical and sedentary behaviour in community-dwelling older adults. Age and Ageing, 40, 205–210. 10.1093/ageing/afq166 21239410

[c33] Matthews C. E., Chen K. Y., Freedson P. S., Buchowski M. S., Beech B. M., Pate R. R., & Troiano R. P. (2008). Amount of time spent in sedentary behaviors in the United States, 2003–2004. American Journal of Epidemiology, 167, 875–881. 10.1093/aje/kwm390 18303006 PMC3527832

[c34] Ng S. W., & Popkin B. M. (2012). Time use and physical activity: A shift away from movement across the globe. Obesity Reviews, 13, 659–680. 10.1111/j.1467-789X.2011.00982.x 22694051 PMC3401184

[c35] R Core Team (2016). R: A language and environment for statistical computing. Vienna, Austria: R Foundation for Statistical Computing.

[c36] Reilly J. J., Penpraze V., Hislop J., Davies G., Grant S., & Paton J. Y. (2008). Objective measurement of physical activity and sedentary behaviour: Review with new data. Archives of Disease in Childhood, 93, 614–619. 10.1136/adc.2007.133272 18305072

[c37] Sellers C., Dall P., Grant M., & Stansfield B. (2016). Validity and reliability of the activPAL3 for measuring posture and stepping in adults and young people. Gait & Posture, 43, 42–47. 10.1016/j.gaitpost.2015.10.020 26669950

[c38] van Uffelen J. G. Z., Heesch K. C., Hill R. L., & Brown W. J. (2011). A qualitative study of older adults’ responses to sitting-time questions: Do we get the information we want? BMC Public Health, 11, 458 10.1186/1471-2458-11-458 21658274 PMC3141448

[c39] Wechsler D. (1998). WAIS-III UK administration and scoring manual. London, UK: Psychological Corporation.

[c40] Wilmot E. G., Edwardson C. L., Achana F. A., Davies M. J., Gorely T., Gray L. J., . . . Biddle S. J. (2012). Sedentary time in adults and the association with diabetes, cardiovascular disease and death: Systematic review and meta-analysis. Diabetologia, 55, 2895–2905. 10.1007/s00125-012-2677-z 22890825

[c41] World Health Organization (2011). Global health and ageing. Retrieved from http://www.who.int/ageing/publications/global_health.pdf

